# A qualitative investigation of lived experiences of long-term health condition management with people who are food insecure

**DOI:** 10.1186/s12889-020-09299-9

**Published:** 2020-08-28

**Authors:** Flora Douglas, Emma MacIver, Chris Yuill

**Affiliations:** 1 School of Nursing and Midwifery, Robert Gordon University, Aberdeen, Scotland; 2 School of Applied Social Sciences, Robert Gordon University, Aberdeen, Scotland

**Keywords:** Household food insecurity, Food poverty, Chronic health conditions, Long-term health conditions, Self-management, Self-care, Support for self-care, Lived experiences, Qualitative research

## Abstract

**Background:**

As more people are living with one or more chronic health conditions, supporting patients to become activated, self-managers of their conditions has become a key health policy focus both in the UK and internationally. There is also growing evidence in the UK that those with long term health conditions have an increased risk of being food insecure. While international evidence indicates that food insecurity adversely affects individual’s health condition management capability, little is known about how those so affected manage their condition(s) in this context. An investigation of lived experience of health condition management was undertaken with food insecure people living in north east Scotland. The study aimed to explore the challenges facing food insecure people in terms of, i. their self-care condition management practices, and ii. disclosing and discussing the experience of managing their condition with a health care professional, and iii. Notions of the support they might wish to receive from them.

**Methods:**

Twenty in-depth interviews were conducted with individuals attending a food bank and food pantry in north east Scotland. Interview audio recordings were fully transcribed and thematically analysed.

**Results:**

Individuals reporting multiple physical and mental health conditions, took part in the study. Four main themes were identified i.e.: 1. food practices, trade-offs and compromises, that relate to economic constraints and lack of choice; 2. illness experiences and food as they relate to physical and mental ill-health; 3. (in) visibility of participants’ economic vulnerability within health care consultations; and 4. perceptions and expectations of the health care system.

**Conclusions:**

This study, the first of its kind in the UK, indicated that participants’ health condition management aspirations were undermined by the experience of food insecurity, and that their health care consultations in were, on the whole, devoid of discussions of those challenges. As such, the study indicated practical and ethical implications for health care policy, practice and research associated with the risk of intervention-generated health inequalities that were suggested by this study. Better understanding is needed about the impact of household food insecurity on existing ill health, wellbeing and health care use across the UK.

## Background

As an increasing number of people are living with one or more chronic health conditions, the practice of ‘support for self-management’ has become a key health policy focus both at home and internationally over the last decade [1–3]. Self-management support has been defined as “health care professionals, teams and services (both within and beyond the NHS) work [ing] in ways that ensure that people with long-term conditions have the knowledge, skills, confidence and support they need to manage their condition(s) effectively in the context of their everyday life” [4]. The perceived benefits of self-management include aspirations to enable people to manage their daily lives better, and the same time, optimise their health outcomes, thereby reducing health care costs [5]. In addition, self-care is associated with concepts of patient empowerment, choice and control [6, 7]. Yet, while this approach has had positive outcomes for some people, the concept has not achieved the universal and sustained behaviour change and condition control sought by such policy [8–10].

The primary focus of much of the patient self-management literature has emphasised educational or instructional interventions targeting perceived individual cognitive deficits, and has tended to ignore or downplay social and material or economic considerations in patients’ lives [1, 2]. At the same time, contemporary healthcare policy rhetoric espouses a shift from traditional approaches, where power and authority are held by health care professionals, towards discourses which valorise *person-centredness* and *shared decision-making* [11]. Yet, health care professionals can hold unrealistic expectations about their patient’s resources and capacities to make lifestyle modifications required for optimum condition management, despite holding beliefs they are acting according to patient-centred principles (ibid). Support for self-care from health care professionals has been found in some cases to have had the opposite effect to that intended, i.e. disempowering and undermining as opposed to enhancing patients’ experiences of health care [12, 13].

One material and social challenge facing an increasing number of people in the UK with long term health problems is the experience of household food insecurity. Household food insecurity (HFI) as a concept, is internationally recognised as a negative human experience associated with being unable to acquire or consume an adequate quality and quantity of food in socially acceptable ways, and includes the experience of having anxiety and uncertainty of being able to do so [14]. In high income countries, HFI is increasingly considered to be primarily associated with a person or household having inadequate or insecure access to food due to financial constraints and regarded as indication of economic struggle in an increasing number of low-income households in high-income countries [15–18]. HFI has been shown, in other international contexts, to be associated with an increased risk of serious non-communicable health conditions such as diabetes and cardiovascular disease, and compromised condition management leading to sub-optimal health outcomes [19–22]. In North America, HFI has also been independently associated with increased health care use and costs [23–25].

The United Nations Food and Agricultural Organisation estimated that 9.3% of the UK population was moderately or severely food insecure in 2014–2016 and that this figure was recorded as 5.6% in 2017–2019 [26]. However, current and future projections for people living in poverty in the UK point toward a worsening picture [27, 28]. People living in the most deprived circumstances in Britain record a 60% higher prevalence of long-term conditions than those living in the most affluent circumstances. Health care expenditure associated with long term conditions is significant, representing £7 of every £10 of UK health and social care spending. Fifty percent of all general practitioner appointments are concerned with dealing with people affected by a long-term health condition, and this population group also accounts for 64% of outpatient appointments, and 70% of in-patient hospital cases [29]. In Scotland, over 2 million people (40% of the population) are currently affected by a long-term health condition or conditions [30]. The 2017 Scottish Health Survey (the first UK commumity health survey of its kind asking questions of food insecriy experience) found that 18% of those living with a long term limiting illness were also food insecure [31].

People living with health conditions are also known to be the highest users of food banks in the UK [32, 33]. There is emerging evidence within UK that some health and social care professionals are also referring some patients to a food bank for help with food provisioning [34]. However, the notion that foodbanks, as charitable emergency response-based entities, are in a position to offer a food supply that can sustainably meet wider community demand, and provide sufficient quantities and nutritional quality of the food needed to meet individual patient’s needs, is problematic [35–40]. Seligman et al. (2018) argue that involuntary, constrained food access not only undermines people’s ability to cope with their condition, but the ongoing uncertainty and stress associated with living with food insecurity can lead to (mal) adaptive coping strategies and practices which can lead to poor condition management [41]. For example, hoarding food and overeating when food is available, limiting or reducing the types and amounts of food consumed when the household income is very limited. A UK independent enquiry on food and poverty in 2015 established that people living in poverty actively seek calorie-dense food when shopping, and knowingly and deliberately seek calories over more expensive nutrients to maximise their food budget, as a survival strategy [42]. This is a particular concern given that the intersection of low income and debt with ill-health or disability, is known to increase the risk of destitution.

The experience of food insecurity as a health care issue, and its impacts on patients’ health condition management endeavours, has received relatively little attention in the UK to date. This lack of attention is in contrast to other high-income countries where poverty and food insecurity, and long-term condition prevalence are similarly high [21, 23, 25, 43–48]. A recent study of Scottish-based health care professionals, by the lead author, found that most believed that some of their patients were affected by food insecurity and that it was impacting their ability to manage their health condition(s) [49]. There were particular concerns about medication adherence and side effects, diet dependant conditions and mental health. Nevertheless, the study also indicated that those health care professionals experience practical and ethical uncertainty about how to identify and respond to food insecurity among their patients (ibid). Given what is known about the observed association and negative impact of the experience of food insecurity on health outcomes and health care use (from international evidence), the lack of attention food insecurity has received as a health care issue in Britain to date, and the prominence of self-care in UK health care policy, we set out to explore individual lived experiences of health condition management with people living here who were also food insecure. This paper therefore reports on an analysis of the findings of a study of lived experiences of condition management from people who were food insecure, their experiences of health care professional support, and their expectations the support they would wish to receive.

### Aims

The study set out to investigate A. what challenges face food insecure people affected by a long-term health condition as far as their self-care / management practices are concerned; B. what issues they encountered in disclosing and discussing the experience of managing their health condition with a third party like a health care professional; C. what sort of support they would wish from a health care professional.

## Methods

The study was based in the north east of Scotland where local academics and community-based agencies supporting local food insecure people had identified a growing trend in the numbers of those with long term health conditions who had been or were being helped with feeding by local agencies [50]. This research was therefore developed as a collaborative project between the academic researchers and community-based stakeholders due to their existing relationship and local knowledge of the health challenges facing people living with extreme resource constraints in local communities, and beyond. The study was also informed by two conceptual frameworks: Fram et al’s 2015 Household Food Insecurity Causes and Consequences framework [51] and the Massachusetts Medical Society Cycle of Chronic Disease and Food Insecurity [52].

The principles and techniques found in Grounded Theory approaches guided this work [53]. Semi-structured interviews were chosen to address the research objectives on the basis that so little is known about this emerging public health issue.^1^ An interview topic guide was generated from the study aims and conceptual underpinnings, and then discussed with the research partners to determine its relevance, perceived gaps and preferred questioning language. Two food bank volunteers who had lived experience of food insecurity then also reviewed this draft guide for question relevance and language. The topic guide led participants through a discussion about i. who the participants lived with; ii. what health conditions they were affected by; iii. Their experiences of food security and insecurity in general; iv.; how those experiences played into, (or not) their self-care practices; v. their experiences of engaging health professionals in discussions about any challenges they were facing in this regard; and vi. their thoughts about the help or support they thought people and like them could receive from them that would be helpful to them.

### Study group of interest

People who self-identified as having a physical or mental health condition or conditions and who were using local food banks or food pantry were targeted for recruitment for this research. A combination of purposive and convenience sampling was used to try and achieve a balance of gender, age, and self-reported physical and mental health conditions.

### Recruitment process

Recruitment took place in the partner’s food bank and their recently established food pantry. The food bank was located in the city centre. It issued free food parcels on a rationed basis according to household size, with food parcel items preselected and packed into bags by food bank staff. Their contents were made up according to the foods available from the larger food store and varied from week to week according to the food supplied from external sources; corporate or public donations. All food bank parcel items were confined to those that could be stored at an ambient temperature, i.e. were packet, tinned or bottled food. The food pantry was located in an adjacent local suburb and operated on a local cooperative basis. Here people apply to join as a pantry member and then for £2.50 a week, were able to choose 10 items of food from the pantry according to what was available that week to the food pantry. It was supplied in a similar fashion to the food bank. The food pantry was able to offer items such as fresh milk, cheese, meat and fish due to having cold storage facilities available. These food items were not available in the food bank parcels.

Local staff and volunteers facilitating recruitment to the study were briefed by the researchers before the recruitment period. Posters advertising the study were displayed in both locations. Participants were recruited to the study through two pathways. Staff members flagging the study up with people who they knew had disclosed they had a health condition through their previous dealings with them and thought might be interested and eligible to take part, or, through people making themselves known to staff as wishing to take part because they read the posters. All potential participants were provided with an information sheet about the study and those who agreed to take part were asked to sign a consent form. The study had been reviewed and approved by the School Ethics Review Panel, School of Nursing and Midwifery Robert Gordon University. Participants were also asked to complete a short demographic questionnaire prior to each interview. Twenty individuals (eleven men and nine women) took part, with three men and three women recruited from the food pantry, and eight men and six women from the food bank.

All interviews were carried out by EM in private areas situated within the food bank and the food pantry, and audio-recorded with participants’ consent. The interviews lasted between 15 and 40 min. One lasted for 1 h and 30 mins. Field notes were also created as the study proceeded with the researcher noting observations from her interactions with study participants, and other notable and relevant occurrences during their time in the field. Each person taking part was given a £20.00 shopping voucher in recognition of their time and expertise shared once the interview concluded. During recruitment all members of the research team and with partners were kept up to date with the participant characteristics as people agreed to take part. We aimed to try to have a balance of gender, age and health condition representativeness. The research partners develop relationships and knowledge of the circumstances of many of their clients (because of their work with them - which they were also conscious to protect and respect), and were able, because of this, to flag up the study to range of people they knew were we were keen to engage with for this study. It was through this process of sensitive, purposive sampling that we were able to achieve the demographic and illness profile reported in this paper. Verbatim transcriptions of the interview audio files were produced and checked for accuracy. The interview transcriptions and field notes were the data sources used during the analysis.

### Data analysis

An initial set of seven interview audiofiles, transcripts and field notes were reviewed and were checked and read line by line by both EM and FD. Subsequently, an initial set of descriptive codes were developed according to the issues and constructs observed within them, and those codes were refined as more data was collected and used to code (by EM working in collaboration with FD) the remaining interview transcripts as they were produced. It became clear to us by the later interviews that we were not picking up any additional new information and concluded that we had reached data saturation in respect to the defined study questions we were concerned with. A set of analytic memos was also developed from reflections on the coded interview data and the field notes, and all those data were used to derive the main themes that emerged from our reading and interpretation of the data reported in this paper. The data analysis was supported by the use of NViVo ver11 software to store and help us manage the data. Our reflections on the findings and our initial set of conclusions were shared with the research partners and study participants prior to finalising our analysis. The research partners found that our findings corresponded to their impressions of the challenges their clients faced. At the same time, they expressed some surprise at the extent of ill health reported amongst our study participants. We received no comments back from participants on the written draft feedback we sent them for review and comment. All individuals and their illustrative narrative quotes (where relevant) are represented using a pseudonym.

## Findings

### Description of the sample

People who took part were aged from 26 to 83 years of age (mean age = 53). All but one reported having multiple conditions with 16 participants reporting they were living with three or more conditions. All were on medication and in most cases, multiple types of medication. Thirteen participants were retired or were unable work due to ill-health with the remainder either in paid or voluntary work. Table 1 provides a full account of the participants’ profile.

**Table 1:** Study Participant Profile

Gender	Age Range (in years)	Ethnicity	Self-Reported Health Condition(s)	Employment Status	Self-Rated Health Status
Male	70–79	White Scottish	Stroke, mobility issues, left-sided weakness	Permanently sick or disabled	Good
Male	80–89	Other British	Heart problems, circulation issues, stomach issues	Permanently retired from work	Fair
Female	70–79	White Scottish	COPD, osteoporosis, stomach ulcer, back and leg pain, mobility issues	Permanently retired from work	Fair
Male	50–59	White Scottish	Angina, heart problem, asthma, sciatica, mobility issues	Permanently sick or disabled AND looking after family	Very Bad
Female	70–79	White Scottish	COPD, osteoporosis, asthma, fibromyalgia, heart blockage	Other – volunteer	Bad
Female	40–49	White Scottish	Under-active thyroid, stress following family bereavement	Unemployed and seeking work (volunteer)	Fair
Female	30–39	White Scottish	Polycystic kidney - kidney failure, depression and anxiety	Unable to work due to illness	Bad
Female	60–69	White Scottish	Fibromyalgia, osteoarthritis, spondylitis, asthma, diverticulitis	Unable to work due to illness	Bad
Female	50–59	Irish	COPD, IBS, anxiety and depression	Unable to work due to illness	Bad
Male	40–49	White Scottish	Lower back pain, various injuries to feet and ankles, psoriasis, mental health problems and anxiety, severe migraines, suicidal thoughts, self-harm. Previous history of drug misuse	Permanently sick or disabled	Fair
Male	60–69	White Scottish	“Horseshoe” kidneys (low kidney function), anxiety, IBS, digestive problems, high blood pressure and cholesterol	Unemployed and seeking work (volunteer)	Fair
Male	60–69	White Scottish	COPD, agoraphobia, fibromyalgia	Unable to work due to illness AND Permanently sick or disabled	Bad
Male	50–54	White Scottish	Arthritis and depression	Unable to work due to short-term illness or injury	Fair
Female	30–39	White Scottish	type 1 diabetes, anorexia, depression, anxiety	Employed part-time	Fair/ Bad (varies)
Male	50–59	White Scottish	depression, arthritis, high blood pressure, stomach problems	Unemployed	Fair
Male	20–29	White Scottish	Type 1 diabetes	Employed part-time and volunteer	Good
Male	40–49	White Scottish	Type 1 diabetes, strokes and depression	Other - volunteer	Fair
Female	50–59	Other British	Lupus SLE, underactive thyroid, arthritis, tendonitis, MH issues, endometriosis, irritable bladder, irritable bowel	Employed full-time	Bad
Female	30–39	White Scottish	Anxiety and depression, asthma, arthritis	Permanently sick or disabled	Fair
Male	50–59	White Scottish	Depression, history of substance misuse. Previous Hepatitis C	Unemployed & Seeking Work AND Unable to work due to short-term illness or injury	Good

### Study participant profile

Our analysis generated four key themes which included: 1. food practices - compromises and trade-offs that related to economic constraints and lack of choice; 2. food scarcity and illness experience as they related to participants’ physical and mental ill-health; 3. the (in) visibility of economic vulnerability in the context of health care consultations: 4. participants’ notions of useful health care professional support in relation to their health condition self-care practices and life circumstance challenges.

### Food practices - compromises and trade-offs

Eating was commonly described as an erratic and solitary activity, which provided little enjoyment, or the nutritional balance necessary for good health. Choice and agency over food consumed was severely limited. This dictated not only what participants said they were able to buy or were given to eat by the food bank, but also where and when they were able to eat. For example, this participant talks about being advised by his doctor to stay off work for a few days, which he ignored to acquire to milk for his tea:

*...cause the worst-case scenario is I run out of milk, for my tea … But, er, that’s happened a couple of times and all, and I’m like, right, I know I’m no feeling well, and say the doctor’s telt me to stay off work for a couple of days, I’ll just come into work, just for the sheer fact I get milk...but I’ll do a full day’s work just to get that milk, do you know what I mean?* (Keith, 48).

Few participants ate three meals a day. Most reported eating one meal a day or going without food for several days and living on beverages such as tea and coffee during those times. Some viewed this pattern as their normal, illustrated by this example where the participant talked about his daily routine which was characterised by a lack of predictability of there being food in the house to make even one meal in the day:

*I get up in the morning, maybe have a cup of tea, depending .., sit about, maybe wait to supper time, maybe throw something in the microwave or the oven, if there’s something there, and that’s it* (Pete, 44).

Others thought their diet fell short of what they thought should and wanted to be eating. Jimmy talks here for example, about having to buy and eat cheap, carbohydrate-based food that he wouldn’t ordinarily choose to eat:

*To make it [money] spread further cause you’re buying, you’re buying food you don’t usually, we don’t want to eat, you use it, like pies and things, we’ll three, four in a packet, or cheap pizza which is, you get one for a pound, that’s a meal in itself basically, and er, just cheap kind of loaf …* (Jimmy, 61).

The example above illustrates a coping strategy commonly described across the sample involving the maximising (stretching out) their household food resources by buying cheap or quick and easy to prepare foods.

Other coping strategies described, included cooking from scratch using inexpensive ingredients (such as cheaper cuts of meat which tend to be higher in fat, described in the following quote) and/ or food hoarding for times when money and food may be scarce:

*I tend to do like a, a, a tinned shop. I’ll buy like all the, the beans and spaghetti and pasta and stuff, you know, the dry stuff, I’ll buy all of that sort of monthly and then the rest is kind of weekly, cause I don’t know what money I’m going to have for the month and I don’t know what’s going to happen and, who’s going to need what or, whatever, so it tends to be just weekly picking up, you know, maybe a pack of mince but, we would, like a pound of mince would do maybe three meals for the two of us so, you know, and we would just, I’ve got like packets of mince in the freezer, like half a pack or a third of pack, tend not to, you know, spread things out rather than bulking things up with the mince* (Heather, 54).

It was also evident that people had a clear system of prioritising other family members’ food needs were prioritised above their own. In particular, mothers described ensuring that their children’s nutritional needs were satisfied first. This practice applied to both adult children, as well as dependent, young children:

*… obviously I’ve got to feed the kids. They still are my main priority ...* (Julie, 37).

Additionally, bills, such as housing costs and heating were prioritised and paid for first. Food assumed lesser status as an area of spending and was often minimised to accommodate other necessary household expenditure:

...*and, with my budget, I think food is basically the bottom of the list...pay for everything and then, then it’s food shopping and generally there’s just not really a lot of money for food shopping* (Heather, 54).

All participants expressed appreciation for the support they received from the food bank or food pantry. Both organisations were described as a key source of basic foods (such as tea, coffee, sugar, pasta, jars and tinned foods), as well as fresh produce (including fruit, vegetables, some meat and fish), that would otherwise be out of reach due to budget constraints. They found that both organisations could not always meet all of their dietary needs. That experience was tempered with low expectations that their needs could be met at all. This appreciation for and perception (and acceptance) of food supply constraints is illustrated here where one participant with lactose intolerance maintained:

*They’re (food bank) quite good. If there’s any soya milk, that sort of thing, they keep it back for me … they do a sterling job, yeah …* (Tom, 61).

### Food scarcity and illness experience

All participants were dealing with one or more physical and/or mental health condition. Some participants reported they were living with conditions such as diabetes and bowel problems that required dietary monitoring and management. Diet (quantity and quality) was also a key issue in many other types of conditions because it played an instrumental role in medication regimes, and people’s overall health and well-being.

Those who reported having diabetes or a condition that required care and monitoring of their dietary intake, such as bowel conditions, indicated they had good knowledge of the sorts of foods they believed they should be eating to manage their condition. However, realising that knowledge was not always possible due to financial constraints. Some found affordability was a barrier to an appropriate diet on a regular basis, others, from time to time. Those participants described using a range of coping strategies to help them deal with fluctuations in their household food supplies. These included; skipping meals, cutting back on medication because of food scarcity, adopting a “trial and error” approach to eating potentially troublesome, but affordable foods, and, food hoarding during times when financial constraints were less severe, illustrated in the comments below:

*I might eat something and I’ll feel extremely bloated or extremely tired...* (Mary, 53).

*I could maybe go and buy and say, well, that’s maybe like £2 something, I’ll try that, if it doesn’t work, I’ll know, I canna buy that again...* (Patricia, 66).

*what I try and do is, try and stock up a wee bit so, we’ve always got food in the cupboard, you know, even if it’s tinned stuff*...(Grant, 51).

All participants were taking some form of medication and in most cases, multiple types of medication. Some of those on oral medication, which needed to be taken with food, said it wasn’t always possible to do so because they didn’t always have enough food. Jenny discussed how she regularly missed a dose of her medication to avoid unpleasant gastric side effects:

*my arthritis medication, I’m meant to take that three times a day but, I’ve to take it with food or it can make you quite sick...so I, find that I can only take those tablets twice a day. So, I’m not getting the good of them …* (Jenny, 30).

Indeed, several of the participants appeared unclear about the dietary advice around taking their medication(s), specifically whether medication should be taken with food or on an empty stomach. Some participants questions during the interview concerning medication and dietary advice prompted reflection of the consequences of overlooking stated advice on taking medication.

*… six months ago I was on like 34 tablets a day, ken … and there was only a couple of them I noticed that you’ve got to take with, with food or without food but, I never really look at that … maybe that’s why I sometimes get a sore stomach and everything, ken, cause actually thinking about it* (Pete, 44).

Commonly, people also described how lack of food, lack of choice over food, and/or unappetising food had an adverse effect on their mental health, as illustrated below:

*Well, it definitely affects your health cause, if you have, erm, nothing in the fridge that you would consider nice then you’re just going to not bother and you’re going go back to bed and not eat anything... it will lower your mood …* (Mary, 53).

Eleven of the 20 participants stated they were suffering from depression at the start of interview.

### (In)visibility of economic vulnerability

We asked participants about their experiences of discussing their food access challenges with their health care professionals including their general practitioner (GP).^2^ We were struck by the extent to which their narratives revealed that this issue remained unspoken and seemingly invisible in those discussions. Most believed their GP was unaware of their struggle to put food on the table and commonly indicated it was not a subject that their GP raised with them during a consultation. Many considered GPs to be exclusively concerned with treating their presenting physical or mental illness, as Grant notes:

*It’s just, I get the impression if you go to the doctor, you’re nae wanted there, ken what I mean? You just, you’ve to get in and get out as quick as possible … Well, they’ve got 10 min to get you in and out and that’s it...* (Grant, 51).

This quote also represents a commonly expressed perception that GPs were working within tight time constraints as far as appointments were concerned, and this factor inhibited the opportunity to talk about any possible financial problems that might prevent them following any dietary advice given. GPs were viewed as extremely busy, *as it was*, to talk about this sort of issue:

*the GPs aren’t really interested, they’ve got enough to do that they’re nae going to turn around and say, “well, have you had something to eat today?” …* (Raymond, 56).

It was also notable that GPs were considered to lack the appropriate knowledge and understanding to assist them with this problem:

*my first thought of that would be, do the GPs actually know what’s out there, erm, I’m going to say no … I wouldn’t go to my GP to ask...*(Lucy, 31).

This was not a perception that was confined to GPs. Other types of health care professionals were cited as giving participants advice that was not economically feasible for them to follow either. This lack of awareness or understanding became apparent as people spoke about being advised to eat certain foods or follow a specific diet that was unattainable due to cost, illustrated below:

*… it was like the renal nurse, she told me to try like joining Slimming World but, I can’t afford it* (Julie, 37).

In a few cases, participants described their health care professionals advising and directing them on food management practices they were not able to follow due to the debilitating symptoms and bodily experiences associated with their health conditions. One woman talked about her difficulties when her GP advised on cooking advice during a consultation:

*he told me just like porridge but, make porridge from scratch and like, I don’t have energy to do that...* (Julie, 37).

While most participants did not think that healthcare professionals were aware of their patients’ financial struggles, most also admitted that they didn’t actively disclose or volunteer information about their financial issues or food insecurity to their GP or healthcare professional either.

### Notions of useful health care professional support

When asked what sort of support they wished to receive from health care professionals in relation to being food insecure, most participants thought it would be helpful if those professionals were aware of this challenge. In some cases, participants thought that GPs and other relevant professionals *should* be aware when people were experiencing food insecurity. GPs in particular were viewed as being the first point of contact with primary care services, and thought to have an important role to play in supporting people affected by food insecurity. Some believed it was down to the individual person to reveal this problem, yet at the same time, held the view (along with other participants) that health care professionals should proactively probe to find out if people were struggling to cope. The following quotes illustrates the conundrum revealed by this discussion in the data. In this first example, Jenny talks about a perceived role for GPs signposting to a dietician that she thought might help her cope better with her situation. Initially, she indicates that she should raise the issue with the GP, but simultaneously asserts that this sort of support should be routinely offered by the GP as routine. The second quote also highlights the challenge that flagging financial struggles to health care professional represents to some people with mental health problems, with this participant concluding that there should be more obvious signals coming from the health care environment that this is an issue people can get help with:

*I think that would be something [GP referral to dietician] I could ask for...and then, but it should be something that’s offered* (Jenny, 30).

and

*if you have a certain type of mental health issue it’s hard to do that, asking, the approaching, sometimes, oh, they’re better off with not being here and, if that makes kind of sense so, it can be difficult. I think, I think there should be a lot more information for individuals saying, this is available for you, this is where you can go, this is who can help...* (Susie, 49).

Some participants thought health care professionals themselves would find it difficult to raise the issue because they might be concerned not to offend their patients, illustrated here:

*… they canna really say to someone cause they dinna want to hurt their pride …* (Patricia, 66).

However, the dominant theme in the data was the notion that it should be the responsibility of the health care professional to enquire about financial challenges as it was a difficult topic for patients to raise. This perspective was thought to be due to feelings of shame, embarrassment and in some cases, exacerbated by their health condition, as discussed above.

One dimension of the perceived benefits of a health care professional knowing about a person’s financial struggle was the apparent comfort that would be derived from knowing the GP or health care professional was aware of their position. It seemed that health care professional-knowing (which we believe was something akin to having empathy with their position) would, for some, be sufficient and mentally therapeutic in itself, as explained here

*.. at the end of the day it’s, it all goes back to the same, if, if it gets it out of your head and somebody listened to you and they tell, kind of, it’s just a different, somebody else’s different, looking at it a different way* (Raymond, 56).

The other dimension of this health care professional-knowing was the view that it would enable better access to food-based help and support. Health care professionals were viewed as important signposting or referral agents to services such as food banks, support groups, food pantries or social groups or specialist health and social care professionals and services. Those included dieticians, social workers, nurses, community psychiatric nurses and pharmacists. Participants’ expectations of health care professional support did not include their being able to help with or alleviate the financial challenges that has caused our participants to be food insecure in the first place.

## Discussion

This investigation was motivated by the need to increase understanding of a health issue highlighted by a growing body of UK based foodbank use research as well as concerns expressed by health care professionals in relation to their experiences of providing support for patients suspected to be food insecure [32–34, 49, 54]. To the best of our knowledge, this is one of the first studies of its kind in the UK to ask questions of people with health problems about what it means to live with their condition(s) while living with food insecurity, and about their interactions with health care professionals’ in relation to support for ‘self-care’ offered within this context. The key premises of support for self-care, is that individuals exercise agency, choice and control, and as a consequence, can be active partners or asset bearing co-producers of the solutions to their health problems [55, 56]. A key finding from our work illustrates that notions of choice and control for our participants, over dietary intake is limited, at best. Moreover, this fundamentally important and challenging aspect of their lives does not appear to have be raised or actively discussed within health care consultations discussed in this study.

This research also provides a picture of people affected by multiple health conditions that are striving to cope and manage as best they can, on food resources most believe are not adequate for their general health or their specific health condition needs, due to low income. This finding resonates profoundly with Garthwaite’s 2015 ethnographic study of food bank use and ill health in England which focused specifically on mental health issues, foodbank experience, and negotiating a healthy diet on a severely limited budget [32]. Our participants described using multiple food coping strategies which differed according to the presence, absence, or feared absence of food. For example, shopping carefully and deliberately when money was available and storing food for future use, or stretching out, minimising food intake or going without when food was scarce or absent.

This largely hidden work of coping with food insecurity was also taking place alongside similarly invisible labour undertaken by the majority our participants which was associated with caring for partners, children and / or parents, earning a living or trying to maintain a household income, as well as undertaking the necessary illness work needed to manage their health conditions. It is also only recently the case that we have begun to recognise the effort and energy required to navigate and cope with the impact and reality of illness diagnosis and symptom management in people’s lives [57, 58]. This work is challenging enough for people who are food secure, never mind for people who are also preoccupied and physiologically challenged by a lack of access to a stable and appropriate food supply. The experience of coping with the resource constraints of poverty itself diverts mental energy to those concerns, thereby reducing an individual’s available cognitive capacity to deal with other necessary day-to-day tasks and decision-making [59, 60].

The extent to which participants reported eating on their own and on an infrequent basis was reminiscent of Hamelin’s seminal study conducted in Canada in 2002 on the experiences of food insecurity of low-income households [61]. This work identified food shortage, unsuitability of food and diet, and a preoccupation with maintaining food access for the household. Hamelin’s research also highlighted the burden of psychological distress and sense of social alienation experienced by those living with food insecurity. Our participants also reported experiencing low mood as a consequence of living with food scarcity and unappetising food choices. Some also indicated that they lacked energy or motivation to prepare meals due to their symptoms, and had difficulties with meal preparation due to physical impairment. However, the majority of the participants articulated a clear understanding of what constitutes a healthy diet and specific dietary needs for their particular condition (where this was relevant), but highlighted that the main barrier to eating as healthily as they wanted to was due to lacking the financial means to do so. Just under a half of our study participants also reported some form of pre-existing mental condition, which is in line with observations elsewhere in the UK [32, 62]. Garthwaite’s study also revealed the lack of predictable food supply was commonly reported as an additional challenge for those affected by pre-existing mental health conditions, with the effort and mental energy associated with trying to maintain access to a food supply [32]. High income countries that are able to monitor HFI prevalence have found a clear association with food insecurity and poor mental health outcomes [63–68] including suicidal ideation and substance use problems in young adulthood [63, 69]. Interventions aimed at reducing food insecurity are considered to be an effective approach to preventing or ameliorating a significant burden of mental health problems in the population [48, 69].

The gratitude for food bank and pantry help expressed by the majority of our participants was stark; an observation noted in other studies involving food bank users [50, 70]. However, there seemed resigned acceptance or little expectation that these food sources would be able to supply them with their dietary needs. US research has found that food insecure people living with diabetes are more likely to seek, but not receive, healthy food items from food banks and use coping strategies such as low-cost food purchasing, and, watering down of food and drinks, which are detrimental to good glycaemic control [45].

We talked to a group of people who were affected by multiple conditions. Those with diabetes or a condition requiring dietary monitoring and management indicated they had good knowledge of the sorts of foods they believed or had been told would help them manage their condition, but often couldn’t afford to follow due to cost. This is reflective of the findings of investigations of lived experiences of diabetes management in low income groups in Canada and the US [71, 72]. There is also emerging evidence that Scottish health care professionals are encountering patients who are also striving to manage diabetes through diet alone by decreasing their carbohydrate intake and increasing intakes of healthier alternative foods, but failing to sustain those behaviours (and achieving good glycaemic control) due to food costs and the stress of changes to household income [49].

Yet again, in countries that capture and monitor food insecurity, Type 2 diabetes is known to be more prevalent amongst food insecure groups [71–78], with poverty and food insecurity itself implicated as a determinant of the condition [72, 74, 79]. Indeed, food insecurity is considered a powerful antecedent in the development of many chronic health conditions including visceral fat accumulation [20]. Poorly controlled diabetes is associated with comorbid complications such as hypertension, stroke, kidney failure, retinopathy that can lead to blindness and cardiovascular disease [19, 76, 77, 80]. Furthermore, mortality rates from diabetes are also higher in low income groups compared to less deprived groups affected by diabetes [72]. Food insecurity is a known barrier to optimum glycaemic control for people affected by diabetes [19, 81, 82], but to the best of our knowledge, this issue has received little attention in the UK, and this study suggests that there is an urgent need to do so. In addition, other conditions that require good dietary management and monitoring, for example kidney disease [83] and bowel conditions should also be investigated in relation to food insecure populations.

In relation to food insecurity impacting on medicine use, some participants appeared unclear about the dietary aspects their medication regimes, i.e. whether this should be taken with food or on an empty stomach. Queries in the interview around medication and dietary advice, led to some participants re-examining the consequences of overlooking stated medication advice, leading one individual to wonder if the severe gastric symptoms he had experienced in the past had in fact been associated with his low food intake. There was also some indication that a few participants were taking reduced amounts of their prescribed medication to avoid gastric symptoms. Whilst in Scotland, cost is not generally considered a barrier to accessing medication, it does raise the question about how both food insecurity and medication costs might be playing into the food and medicine taking practices of people living in parts of the UK where this is not the case [82, 84, 85]. Regardless of national context, these findings raise questions about the potential role for those involved in prescribing (medical and non-medical prescribers) in working with people at risk of or who are experiencing food insecurity.

This study also points toward a significant challenge for health care professionals in providing effective, person-centred support for their food insecure patients [1]. Our participants indicated they believed that it was important that health care professionals were aware of theirs (and other patients’) struggles to put food on the table. Nevertheless, most did not believe health care professionals had enough time or relevant knowledge to be able to help. Some were unsure that GPs in particular were interested in anything other than physical ailments, and these perceptions may not have been without foundation. Investigations of health care patient / professional interactions in other sensitive lifestyle areas have also revealed significant barriers to effective and non-judgemental communication. Those include on the patient side, feelings of stigma and powerlessness, and on the health care professional side, professional attitudes, confidence, training, and, available health care contact time [86]. There is also some emerging evidence that health care professionals are not only reluctant to raise the issue of financial challenges with patients but admit to having trouble recognising if someone was struggling to put food on the table [49, 87]. Social distance between health care professionals and patients is also theorised as a barrier to effective and empathetic interaction [88–90]. Some of our participants indicated they viewed health care professionals as ‘other’ in respect of their view of them not knowing what life was like for them. Franklin et al’s qualitative synthesis of patient and professional perspectives on support for selfcare interactions, revealed that the traditional models of healthcare consultation continue to prevail, (i.e. health care professionals operating as authority figures in the interaction) which results in limited opportunities to develop shared understandings of the patient’s social context [91].

Yet, our study participants believed it would help them and others like them to cope better with their health condition, if their health care professionals were aware of the financial challenges that prevented them for managing their health condition. One dimension of the perceived benefit of this was the apparent comfort that would be derived from knowing that their health care professionals were aware of their situation. There was an indication here that some people perceived empathetic conversations about financial challenges to be the most useful support they could receive for health care professionals; with no expectation that they would be able to do something to address this issue for them. However, here too there is a challenge for health care professionals in determining the best course of action, as there was a general reluctance to raise financial difficulties with health care professionals amongst study participants, with in some cases, apparently deliberate care being taken to hide their poverty from public view. A few participants felt strongly that it was down to the individual person to reveal this problem to their health care professional, while others thought that health care professionals should proactively probe to find out about financial struggles because they believed it was difficult for people to do this themselves. Further work is therefore required to understand how best to support patients and professionals in this area, for it was also clear that our participants not only valued the possibility of more empathy and understanding from their health care professionals but could also imagine that this would get them access to practical support and appropriate advice. Interestingly, none talked about looking for support in gaining more access to financial resources – the root cause of their food insecurity – and is something that we suspect could be a barrier on the part of health care professionals to raising the issue, who may feel powerless to do much about it [92]. This particular finding (*the invisibility of financial vulnerability within the health care consultation*) challenges orthodox notions of self-management support policy and practice within health care settings (in UK and internationally). For it is well established that effective support for self-management requires attention not only to individuals’ medical needs but also to their social, emotional and psychological needs too [91, 93]. Within the UK context and internationally, supporting self-management policy incorporates the need to support patients to, amongst other strategies, improve lifestyle behaviours and access community services [94]. Data suggest that whatever conversations were taking place around these issues between health care professionals and our participants; they were not grounded in discussions cognisant of the lived social and economic realities of those concerned, and therefore seemed to place those individuals at higher risk of feeling more disempowered than empowered by those conversations. One potential way of overcoming the difficulty of raising and discussing financial difficulties in clinical consultations might be through the introduction of a financial screening question within routine clinical practice, as has been muted in other high-income countries [95–97]. This suggestion was raised by health professionals interviewed in a separate study by FD, but it was also pointed out there may be practical difficulties and unintended negative consequences that would require careful testing prior to any wholesale adoption in practice [49].

Fundamentally there is an urgent need to rethink public policies that are driving up the numbers of people affected by poverty and food insecurity in the UK [27, 97–99]. Healthcare professionals can also play a role by recognising and supporting patients with chronic health problems, and resource needs, such as poverty and food insecurity [59, 100, 101].

### Study limitations and strengths

The study has four main limitations. Firstly, our recruitment strategy involved sampling from food insecure groups who were users of a community food bank and food pantry. This was in keeping with the partnership ethos and rationale that underpinned the study as explained above. However, food banks are not used by everyone who is food insecure. For example, Pilkington and colleagues noted in their research with low income people affected by diabetes in Canada, that the majority of those who took part in their study rarely, or never used food banks because they disliked the way they were treated, did not like or trust the food they were given, or found the food they were offered was unsuitable for their needs [45]. Therefore, it is highly likely that eligible participants in the local community did not get the opportunity to take part. It is conceivable that participants recruited to the study through a health care centre route for example, may have had held more critical views of food banks as a source of food, or found it more difficult to be as candid about their encounters with health care professionals than we found amongst our participants. Secondly, the relatively small number of participants involved in this study also means that we make no claims about the distribution of perspectives and experiences reported in this paper. However, we set out to gain viewpoints from as varied a sample of participants as possible, e.g. recruiting people with physical and mental health issues, those who had conditions that typically required dietary modification and monitoring for optimum management, and those who did not, and succeeded in gaining the diversity of experiences we hope to achieve. However, we did not speak to anyone who disclosed a cancer diagnosis and this is an important and potentially overlooked issue as the association between food insecurity and cancer outcomes remains poorly understood [45]. Thirdly, we relied on participants’ self-reported diagnosis and disclosure to make judgments about our sampling strategy and recruitment. However, we have no reason to doubt their accounts of ill health given the detailed experiences shared during the interviews. Fourthly, the experience of food insecurity can change over time; people can be food secure at the start of the month but food insecure at the end of the month or next month, or next year depending on their circumstances. In order to gain a better understanding of impact and responses to fluctuating or changing patient circumstances on health outcomes and health care use, longitudinal studies are needed.

In terms of other key strengths to note, the study was developed due to common concerns of both local community-based stakeholders and advocates, and, academic researchers about the lack of attention paid to this issue in the UK. Furthermore, this co-produced knowledge has resonated with those health, social care and third sector participants both locally and nationally as we have shared and discussed the findings at various knowledge exchange events thus far. All of the above mean we believe that our findings are indicative of issues that warrant further specific attention.

## Conclusions

There is a need to identify how healthcare professionals can help recognise and support patients with chronic health problems, and resource needs, such as poverty and food insecurity. Without attention to this issue during health care consultations, there remains the risk that well intended, but narrow, decontextualized notions of support for self-care could potentially aggravate social inequalities in experiences of health care and wellbeing.

Household food insecurity is not only a serious social and public health concern in the UK, but is also a neglected healthcare issue in need of urgent attention. Whilst time constraints on GPs and other health care professionals are recognised, there has to be wider recognition that people experiencing food insecurity may also be struggling to manage their health condition due to resource constraints that has led to food insecurity, and are doing so without the knowledge and appropriate support of their health care professionals. Consequently, this study indicates a range of practical and ethical implications for policy, practice and research associated with health care professional support for self-care. It also points to the need to understand the extent to which experiences of household food insecurity are impacting on health outcomes, self-care practices and capabilities, and health care use by those affected by long term conditions, and ultimately, the costs the health care system and budget that flow directly from household food insecurity. This work may also help to stimulate some debate within the health care professions, and policy making spheres about the notion of raising questions, within routine health care consultations, about the availability (or otherwise) of the social and economic resources needed to pursue the lifestyle behaviours considered critical to effective self-care for many. This may benefit not only those living within the UK context, by enabling more informed and relevant support for food insecure patients, but within other similar international contexts that are affected by growing numbers of people who are not only living with long term conditions, but also where social and economic disadvantage is affecting growing numbers within their populations in both observable, but also hidden ways.

## Data Availability

The datasets generated and/or analysed during the current study are not publicly available due the fact that participants were not asked to give consent for their data to be made available to anyone beyond the research team, but are available from the corresponding author on reasonable request.

## Abbreviations

HFI: Household food insecurity
UK: United Kingdom

## Footnotes

1 FD (the lead, female researcher) is a Reader who has a back ground in nursing, but is also a public health scientist. EM is a Research Fellow who is a female social scientist with a background in social work and CY is a Senior Lecturer, male sociologist. All are trained and experienced social science researchers with research interests and a track record of research in health and social inequalities, household food insecurity and carer’s experiences.

2 A general practitioner in the UK is a general medical practitioner that is based in the primary care setting, sometimes referred to as a family doctor in other national jurisdictions.
